# PREVENTING ELDER MISTREATMENT THROUGH A CAREGIVER-FOCUSED INTERVENTION: THE COACH PROGRAM

**DOI:** 10.1093/geroni/igad104.0611

**Published:** 2023-12-21

**Authors:** Kathleen Wilber, Julia Rowan, Jeanine Yonashiro-Cho, Laura Mosqueda, Anthony Hou, Zach Gassoumis

**Affiliations:** University of Southern California, Los Angeles, California, United States; University of Southern California Keck School of Medicine, Los Angeles, California, United States; University of Southern California, Los Angeles, California, United States; Keck School of Medicine of the University of Southern California, Los Angeles, California, United States; Kaiser Permanente, Los Angeles, California, United States; University of Southern California, Los Angeles, California, United States

## Abstract

Elder mistreatment has negative implications for individuals, families, communities, and society as a whole, yet little research exists on interventions or prevention approaches. This study reports on the results from the pilot test of an elder mistreatment prevention program. The Comprehensive Older Adult Caregiving Supports (COACH) program was developed as a strengths-based person-centered caregiver support intervention, built on evidence-based prevention approaches used in other types of family violence. In a double-blind randomized controlled trial, family caregivers (n=80) of older adults aged 65 and older who were members of Kaiser Permanente completed surveys at baseline, post-test, and three-month follow-up. The primary outcome was elder mistreatment; additional proximal outcomes were caregiver burden, quality-of-life, anxiety, and depression. All analyses were conducted using nonparametric tests. The treatment group had no mistreatment at 3-month follow-up, a significantly lower rate than the control group (treatment=0%, control=23.08%, p=0.010). Among the proximal outcomes, a significant difference in social quality-of-life was seen at post-test but did not persist to the 3-month follow-up. No further proximal outcome effects were detected. This pilot program offers a promising model to reduce elder mistreatment and its resultant impacts on the health and well-being of both caregivers and the older adults they care for. In helping to ameliorate mistreatment, COACH supports individuals providing crucial care to older adults with chronic health conditions and reduces the burden of addressing this complex condition for an already strained geriatric health and social service delivery system.

